# Pediatric precision sleep network: a study protocol for identifying sleep signatures of mental health risk in peri-adolescents

**DOI:** 10.1186/s12887-026-07253-z

**Published:** 2026-07-03

**Authors:** Amanda E. Baker, Rebecca E. Cooper, Daniel J. Buysse, Alyna T. Chien, Raul Gonzalez, Jessica C. Levenson, Timothy Miller, Sara Rivero-Conil, David Seo, Shyam Visweswaran, Yanshan Wang, Allison Caswell, Simey Chan, Kelly Chiu, Mary Corcoran, Susan Churchill, Ronna Currie, Maurice Duque, Isabelle Elder, Josefina Freitag, Saurabh Jadhav, Rebecca Locke, Christopher Martin, Maria Milla, Rashmi Sahasrabudhe, Soumya Sathe, Karoline Shellhause, Aishwarya Sritharan, Jerrilyn Robles, Annette Werner, Meredith J. Wallace, Maria Jalbrzikowski, Dana L. McMakin, Adriane M. Soehner

**Affiliations:** 1 Department of Psychology, Florida International University, 11200 S.W. 8th Street, Miami, FL 33199 USA; 2 University of Pittsburgh School of Medicine, Pittsburgh, PA USA; 3 Boston Children’s Hospital, Harvard Medical School, Boston, MA USA; 4 Nicklaus Children’s Hospital, Miami, FL USA

**Keywords:** Sleep health, mental health, peri-adolescence, predictive modeling, pediatric primary care, electronic health records, risk stratification

## Abstract

**Background:**

Peri-adolescence (ages 10–13) is a sensitive—and clinically critical—developmental window for the emergence of psychiatric symptoms, yet scalable strategies for early risk detection in pediatric primary care (PPC) remain limited. Sleep disturbances are among the most prevalent, predictive, and modifiable indicators of youth mental health, but current pediatric assessments rely heavily on subjective, single-source reports that fail to capture the multidimensional nature of sleep health. The Pediatric Precision Sleep Network (PPSN) is a longitudinal, multi-site study designed to integrate multimodal sleep data with longitudinal clinical outcomes to improve early identification of psychiatric risk during peri-adolescence—a period marked by rapid changes in sleep–circadian biology and vulnerability to psychopathology.

**Methods:**

PPSN will enroll 1,200 youth ages 10–13 across three metropolitan areas (Pittsburgh, Boston, Miami). Over three years, participants will complete multimodal sleep assessments—including self-report, daily sleep logs, actigraphy, ambulatory electroencephalography (EEG), and passive smartphone-based monitoring—administered primarily at home to maximize ecological validity and reduce burden. Psychiatric symptoms and functioning will be assessed biannually via online surveys and electronic health records (EHRs), including structured fields and unstructured notes processed with natural language processing to extract sleep-related information. Scalable, open-source pipelines will automatically derive sleep features. Analyses will: (1) identify multivariable “sleep signatures”—defined as empirically derived patterns of sleep features across modalities—using factor and cluster methods; (2) evaluate their predictive utility for transdiagnostic mental health outcomes using stepwise machine learning approaches; and (3) examine developmental and contextual moderators (e.g., puberty and sociocultural factors). PPSN will also partner with the NIMH Individually Measured Phenotypes to Advance Computational Translation in Mental Health (IMPACT-MH) Data Coordinating Center to standardize procedures, ensure rigorous quality control, and disseminate open-source analytic tools for the broader research community.

**Discussion:**

By integrating ecologically valid, longitudinal sleep monitoring with EHR-based and survey outcomes, PPSN aims to identify developmentally sensitive sleep signatures that could be translated into scalable screening tools for PPC. Embedding sleep-informed algorithms into primary care could improve precision and equity of early risk detection and inform future efforts aimed at earlier identification and monitoring of mental health risk in peri-adolescence.

## Background

Peri-adolescence (ages 10–13) is a sensitive—and clinically critical—window for the emergence and intensification of psychiatric symptoms, making it a key period for early mental health detection and intervention. Half of all mental health conditions emerge by age 14, and by age 15, mental illness contributes more to disability than any other medical condition in the United States [1–3]. Anxiety disorders often peak during this stage, followed by a developmental cascade of transdiagnostic psychopathology, including depression, substance use, and psychotic disorders in later adolescence and young adulthood [2]. These trajectories highlight the urgent need for early detection strategies that extend beyond disorder-specific approaches. Identifying risk factors that are common, causal, and modifiable is critical to developing transdiagnostic screening algorithms that capture the broadest range of at-risk youth and enable timely, preventive intervention [4].

Sleep is one such powerful risk factor. Disturbed sleep is common in youth—with over half of adolescents failing to meet recommended sleep guidelines and 20–30% meeting criteria for a sleep disorder [5, 6]—and is even more prevalent among racially and ethnically minoritized youth [7, 8]. Sleep disturbances predate and predict the onset of nearly all major psychiatric disorders, with especially robust relationships in peri-adolescence [9–11]. Experimental and intervention studies further confirm sleep as a causal factor: disruption of sleep health worsens psychiatric symptoms, while interventions that improve sleep reduce symptom severity and recurrence risk [12–14]. Together, this evidence establishes sleep as a highly prevalent, causal, and modifiable risk factor with strong potential to support early identification and prevention.

However, identifying which aspects of sleep confer the greatest psychiatric risk remains a central challenge. Sleep disturbances relevant to psychiatric outcomes rarely map neatly onto diagnostic categories such as insomnia, hypersomnia, or circadian rhythm disorders [15–17]. Instead, the ‘multidimensional sleep health’ framework offers a more comprehensive lens, encompassing a broader spectrum of sleep behaviors including duration, timing, regularity, efficiency, alertness, and satisfaction [18, 19]. Our group and others have validated this framework using self-report and actigraphy [19–21], with recent pediatric extensions adding a “behavior” dimension reflecting bedtime routines, electronic use, and parental involvement [22]. Additional features of sleep—including sleep architecture and microarchitecture derived from electroencephalographic (EEG) studies, persistent sleep complaints, medication use, and disordered sleep—also provide critical insight into psychiatric vulnerability [5, 23, 24]. Collectively, this work suggests that risk is better captured by multidimensional *sleep signatures*—individualized constellations of sleep characteristics—than by isolated sleep symptoms alone.

Recent technological advances now make it possible to capture multidimensional sleep health in ecologically valid, real-world contexts. Historically, most large-scale studies relied on surveys, diaries, or observer ratings, capturing only a limited subset of sleep behaviors. A meta-analysis of 25 longitudinal datasets (> 45,000 adolescents) found that sleep disturbances were associated with increased odds of first-onset mood or psychotic disorders in adolescence and early adulthood (OR = 1.88, 95% CI 1.67–2.25), with stronger associations observed for disturbances meeting diagnostic criteria and consistent effects across subjective, observer-rated, and objective sleep measures [11]. Notably, while self- and caregiver-reported sleep disturbances showed consistent associations (ORs ~ 1.5–2.5), prediction accuracy improved substantially when biobehavioral sleep measures—such as actigraphy or polysomnography—were incorporated [11]. At the same time, the authors emphasized substantial heterogeneity in sleep definitions and highlighted the need for improved screening strategies and longitudinal assessment of circadian and behavioral sleep features in youth.

Wrist actigraphy is the most widely used and validated method for continuous, low-burden monitoring of sleep and circadian rhythms. With wrist actigraphy, we can quantify sleep duration, efficiency, regularity, timing, as well as 24-hour rest–activity rhythms. From wrist-worn activity monitoring, irregularity emerges as a robust predictor of psychiatric risk [22, 25]. To support scalability, our team has developed automated pipelines (e.g., *actiSleep*) that integrate diary, light, and accelerometry data for efficient and reproducible sleep scoring [26]. Wearable EEG extends sleep measurement to the neurophysiological level, capturing sleep stages and microarchitecture in naturalistic settings with growing concordance to laboratory PSG [27, 28]. Multi-night, home-based recordings are now feasible, yielding markers sensitive to neurodevelopmental changes [23, 24, 29, 30] and neural circuit dysfunctions implicated in psychiatric risk [31–33]. Smartphone sensing provides a complementary modality and uses accelerometry, usage, and GPS data streams to model sleep and pre-sleep behaviors [34–36]. With smartphone ownership rising from ~ 25% in peri-adolescence to nearly universal levels by mid-adolescence [37], smartphones provide scalable opportunities to capture sleep and contextual factors (e.g., device use, light exposure) in real-time. Combining actigraphy, wearable EEG, and passive smartphone sensing with self-report and EHR data will support the derivation of individualized, multimodal sleep signatures that capture risk-relevant variation across multiple dimensions of sleep health and may improve identification of youth at elevated mental health risk beyond existing screening approaches.

Maximizing the clinical utility of sleep signatures requires their integration into pediatric primary care (PPC)—the most scalable setting for early risk detection, with 85–90% of U.S. children attending annual well-child visits [4, 38]. Yet current EHR-based predictive models for mental health outcomes achieve only modest accuracy, in part because they rely heavily on structured fields (e.g., International Classification of Diseases [ICD] codes) that underreport psychiatric and sleep-related problems by up to 90% [39, 40]. Sleep is particularly under-represented in pediatric primary care: fewer than half of pediatric sleep disorders are captured in structured EHR data [5, 41, 42], pediatricians receive on average less than four hours of formal training in sleep medicine [43, 44], and caregivers often fail to raise sleep concerns during annual well-child visits [45, 46]. As a result, many at-risk youth are missed—particularly those with subthreshold problems that may forecast worsening psychopathology [47–49]. Much of the rich clinical information resides instead in unstructured notes, where manual review identifies substantially more sleep problems than ICD codes alone [50]. Novel machine learning approaches, such as natural language processing (NLP), can extract these features from unstructured data at scale and integrate them into predictive models [51, 52]. Embedding multidimensional sleep signatures into EHR-based algorithms could substantially improve sensitivity, precision, and equity in early risk detection [53–56] by allowing pediatric primary care to leverage routinely collected sleep information as a scalable tool for identifying at-risk youth and potentially guiding developmentally timed preventive interventions.

### Study objectives

**Fig. 1:**
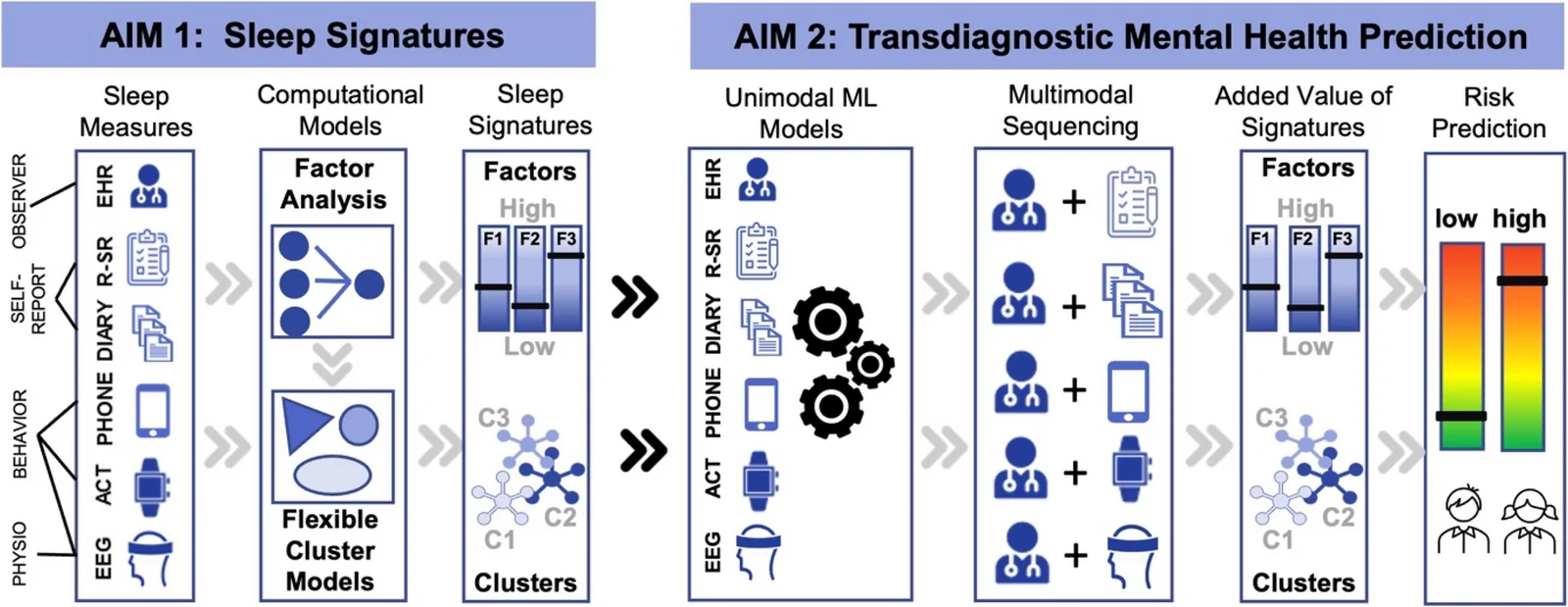
Overview of study aims and analytic approach. Aim 1 focuses on identifying multidimensional sleep signatures using data from actigraphy, EEG, smartphone sensing, self-report, and EHR. Factor analysis will derive latent sleep dimensions, while flexible clustering approaches will identify individual sleep profiles. Aim 2 uses these sleep signatures to predict transdiagnostic mental health risk. Unimodal machine learning (ML) models will be trained on each data source, followed by multimodal sequencing and modeling. The incremental predictive value of derived sleep signatures will be tested, with the goal of developing scalable risk stratification tools for pediatric primary care

The primary aim of the Pediatric Precision Sleep Network (PPSN) is to establish sleep signatures—defined as multivariate combinations of sleep features derived across modalities—that are sensitive to age- and pubertal-related developmental variation and evaluate their ability to predict mental health outcomes in peri-adolescence (Fig. 1). Specifically, Aim 1 is to identify and validate multidimensional sleep signatures by integrating actigraphy, EEG, smartphone sensing, self-report, and EHR data, and to examine how these signatures evolve across peri-adolescence in relation to transdiagnostic psychiatric symptoms. Aim 2 is to develop and validate predictive algorithms for early mental health risk, beginning with sociodemographic, clinical, and EHR variables and testing the incremental value of individual sleep modalities, multimodal combinations, and derived sleep signatures. Aim 3 is is an infrastructure and dissemination aim conducted in partnership with the NIMH Individually Measured Phenotypes to Advance Computational Translation in Mental Health (IMPACT-MH) Data Coordinating Center (DCC). This aim focuses on harmonizing assessments across sites, implementing scalable analytic pipelines, expanding sleep-processing tools, and preparing multimodal data for open sharing via the NIMH Data Archive. Secondary objectives include examining associations between sleep signatures and EHR-based diagnoses, functional outcomes, and developmental moderators, as well as using NLP-derived EHR features to evaluate provider practices and guide referrals for behavioral sleep screening.

## Methods/design

### Study overview

PPSN is a multi-site longitudinal study designed to identify multimodal sleep signatures that predict transdiagnostic mental health risk during peri-adolescence. PPSN will recruit 1,200 youth ages 10–13 across three U.S. sites (Pittsburgh, Miami, and Boston), integrating multimodal sleep assessments—including actigraphy, wearable EEG, smartphone-based sensing, self-report, and structured and unstructured EHR data—with psychiatric outcomes from surveys and structured EHRs. By leveraging accessible, wearable technologies and applying advanced computational methods, PPSN aims to generate pragmatic, scalable screening algorithms for pediatric primary care. PPSN recruitment materials and data collection protocols were designed in consultation with a Community Advisory Board (CAB) to ensure study procedures are relevant, feasible, and accessible for participants and their families across sites. This study is conducted in collaboration with the IMPACT-MH Data Coordinating Center (DCC), which provides centralized data harmonization, quality monitoring, and support for multi-cohort integration.

### Participants

Eligibility criteria are listed in Table 1. Participants will be recruited through primary care practices, practice-based research networks, and established research participant registries across the three participating sites. Collaborators and research assistants embedded at primary care sites will directly recruit youth during their annual well-child visits, facilitating in-person engagement and allowing families to learn about the study in familiar clinical settings. As of manuscript submission, recruitment for PPSN had begun (June 2025) and remains ongoing.

**Table 1:** Eligibility Criteria

Inclusion Criteria
a) Ages 10–13 inclusive
b) Has capacity to comprehend study procedures in English
c) Legal caregiver able to provide informed consent (provided in English at Pittsburgh, and both English and Spanish at Miami and Boston)
d) Actively involved in primary care (most recent well-child visit within 12 months) in the local site’s medical systems

**Exclusion Criteria**
a) Organic sleep disorders or serious or unstable medical conditions
b) Major Diagnostic and Statistical Manual of Mental Disorders (DSM-5) psychiatric disorders, including depressive, bipolar, psychotic, post-traumatic stress, obsessive compulsive or panic disorders. Participants with psychiatric disorders with typical age of onset prior to 10 years (including attention deficit hyperactivity disorder and most anxiety disorders (2)) will be allowed to participate
c) Neurodevelopmental disorders or learning disorders if they significantly impact the ability to understand or engage fully in study procedures

Potential participants meeting eligibility criteria based on their EHRs can also receive MyChart alerts (electronic invitations notifying potential participants of study eligibility) at the Boston site or personalized recruitment letters at the Pittsburgh site. At the Miami site, recruitment will occur through pediatric primary care clinics at Nicklaus Children’s Hospital. Research registries at the University of Pittsburgh (Pitt + Me research registry, https://pittplusme.org) and Boston (Precision Link Biobank [57]) sites together contain over 300,000 participants, and potential participants meeting eligibility criteria will be directly contacted to notify them of the study and to invite participation.

To enhance accessibility and generalizability, recruitment materials were written at approximately a fourth grade reading level and pilot-tested with caregivers to ensure clarity and comprehension across a range of educational backgrounds. Study visits emphasize home-based data collection and flexible scheduling to reduce participant burden related to transportation, time constraints, and competing family demands. Given the linguistic composition of families served at the Miami and Boston sites, study materials and recruitment procedures are available in both English and Spanish to accommodate family language preferences and reduce language-based barriers to participation. Across sites, recruitment strategies are designed to be scalable, adaptable, and reflective of routine pediatric primary care populations, supporting enrollment of a broadly representative and generalizable sample of youth served in U.S. health systems.

### Community advisory board

PPSN includes a Community Advisory Board (CAB) composed of caregivers, youth, clinicians, and community stakeholders representing participating sites. The CAB provides ongoing input on recruitment and retention strategies, study procedures, and participant-facing materials to support clarity, feasibility, and accessibility for families across sites. CAB feedback informs the development and refinement of recruitment materials (e.g., readability and language accessibility), study workflows, and participant communications, and also contributes to the interpretation and dissemination of study findings.

## Measures

### Sleep assessments

#### Wrist actigraphy

Participants will wear the GENEActiv Original (ActivInsights Ltd., Cambridgeshire, UK), a wearable wrist monitor that continuously collects data on activity, ambient light, and skin temperature. Data are collected at 50 Hz. The GENEActiv actigraph has been validated for estimating physical activity and sleep patterns in free-living conditions in peri-adolescents [58]. Semi-automated R pipelines (including *actiSleep* [26], GGIR [59], nParAct [60] and RAR [61]) will be used to clean data and derive outcome variables, including sleep estimates and rest-activity rhythms.

#### Sleep electroencephalography (EEG)

Participants will wear the CGX Patch EEG system (CGX Systems, San Diego, United States), a minimally invasive, portable, multi-channel EEG device that adheres to the forehead. The CGX Patch has been validated in accordance to the gold-standard PSG [28]. The system includes three semi-solid gel electrodes (two frontal, one reference) to record cortical activity during sleep, enabling the ability to derive key neurophysiological markers including slow-wave sleep, rapid eye movement sleep, spectral power, and sleep spindles. Data are stored locally on a MicroSD card and will be uploaded to a secure server upon study completion. We will process the sleep EEG data using open-source tools (e.g., lunaR; https://zzz.bwh.harvard.edu/luna/*).*

#### Passive smartphone sensing

Participants will install the Effortless Assessment Research System (EARS) smartphone application [62, 63], a digital phenotyping tool designed to passively track behavioral and environmental factors. EARS will continuously collect geolocation data, phone usage metrics, accelerometer-based movement data, battery status, and keyboard input. Participants and guardians are asked about rules regarding device usage (e.g. not allowed to have phone at school) to account for periods of limited device access. EARS uses a dedicated keyboard to collect all keyboard input data, except for passwords and information typed into “secure fields.” EARS will also be used to collect daily screen time data and administer a brief self-report survey. The EARS application can be downloaded on both iOS and Android devices, runs in the background, and is optimized to minimize battery consumption, ensuring minimal participant burden. All collected data are encrypted and stored on a secure cloud-based platform (Amazon AWS), ensuring compliance with privacy regulations. EARS will be used primarily to estimate sleep behavior, with secondary analyses examining associations with other behavioral data collected via the app (e.g., mobility, phone use, and daily activity patterns).

#### Daily sleep log

Participants (and/or a caregiver, depending on level of independence) will complete a web-based sleep log each day of the sleep monitoring period, modeled on the Consensus Sleep Diary [64] and adapted for children and adolescents. A survey link will be sent to participants and/or caregivers each morning within an hour of waking. The sleep log will track key sleep parameters (e.g., number and duration of naps, caffeinated drink intake, bed and wake times, night awakenings, perceived sleep quality, perceived alertness, and mood upon wakening) and complement data collected through wearable devices (included in Supplement). Parent/caregiver assistance with reporting is assessed as a part of the sleep log.

#### Sleep questionnaires

Youth and caregivers will complete a comprehensive survey battery of validated and customized questionnaires to assess sleep complaints, habitual sleep patterns, and key factors influencing sleep (see Table 2). To capture subjective sleep symptoms, participants will complete the Patient-Reported Outcomes Measurement Information System (PROMIS) Pediatric Sleep Disturbance and Sleep-Related Impairment short forms [65], which provide reliable, developmentally appropriate indices of perceived sleep quality and daytime dysfunction. Researchers will administer a modified version of the Munich Chronotype Questionnaire (MCTQ [66]), adapted to incorporate online work/school days, to account for greater complexity in sleep-wake behaviors in the post-COVID era (full version in Supplement). Researchers will administer the MCTQ at baseline and at annual follow-ups to minimize clock time (e.g., AM/PM) errors in data input, and youth will complete it individually at 6-month follow-ups. Participants will complete a modified version of the Sleep Environment Inventory [67], adapted for the peri-adolescent population. To further characterize the participant’s sleep environment, caregivers will answer two questionnaires newly created for PPSN: the Household Synchronicity Questionnaire, which measures alignment in sleep schedules among household members, and a Youth Sleep Enhancements questionnaire to document prior interventions or treatments related to the adolescent’s sleep (see Supplement for full instruments).

**Table 2:** Sleep features collected as part of PPSN

Source	Description	Features of interest
Wrist actigraphy (GENEActiv Original)	Worn continuously for 14 days	Daily regularity of sleep, 24-hr rest-activity and light rhythms
Ambulatory Sleep EEG (CGX Patch)	Worn for 7 consecutive nights	Daily regularity of sleep architecture, NREM/REM sleep stages, spectral features, spindles and slow waves
Surveys	PROMIS-Sleep Disturbance and Sleep-Related Impairments (self- and caregiver-report)	Sleep disturbances
	Munich Chronotype Questionnaire (researcher-administered at baseline and yearly follow-ups, self-report at 6-months)	Chronotype, sleep timing
	Sleep Disturbance Scale for Children (caregiver-report)	Sleep disturbances
	Nighttime Parenting Scale	Parenting rules around sleep
	Household Synchronicity	Household sleep patterns
	Youth Sleep Enhancements	History of sleep treatments
Daily sleep log	Completed daily over 14-day sleep monitoring period	Daily regularity of sleep timing and quality, perceived alertness and mood upon wakening, caffeinated drink and psychoactive substance intake
Smartphone application (EARS)	Monitoring of behavioral patterns (geolocation, acceleration, motion detection, battery status, phone usage, keyboard input) associated with mental health, including sleep, for 14 days continuously (*note*: only acceleration and motion detection are required for participation)	Daily regularity of sleep and pre-sleep behaviors
EHR	Sleep disorder diagnosis codes from structured EHR data fields	ICD sleep disorder diagnosis (G47.X) PheCodes
	Sleep features identified using natural language processing algorithm, *nlp4sleep*	Habitual sleep health (e.g., regularity, satisfaction, alertness/sleepiness, timing, efficiency, duration), sleep disorder symptoms, sleep behaviors (e.g., use of electronic media before bed)

*Abbreviations*: *EARS* Effortless Assessment Research System, *EEG* electroencephalography, *EHR* Electronic Health Records, *ICD* International Classification of Diseases, *NREM* non-rapid eye movement, *REM* rapid eye movement

#### Electronic health records (EHRs)

Study team members will access EHRs and collect structured (e.g., demographics, diagnosis codes, procedures, medications) and unstructured (e.g., clinical notes, discharge summaries) data. We will extract structured EHR data elements and normalize them to standardized terminologies. We will map ICD diagnosis codes to established “PheCodes”, or umbrella disease categories, to create clinically meaningful phenotypes for use as input into predictive models [68]. To enhance the detection of sleep-related phenotypes from unstructured clinical notes, we will extend and adapt an existing NLP algorithm, *nlp4sleep* [69], a validated algorithm originally developed to extract sleep health information from unstructured EHR data in patients with Alzheimer’s disease. First, all participating sites across the network will collaboratively develop a comprehensive sleep phenotype concept set, informed by the prior Alzheimer’s-focused work. Based on this shared concept set, the University of Pittsburgh will use UPMC’s clinical notes to construct a gold standard corpus of pediatric encounter notes through manual annotation by clinicians and trained researchers. This corpus will be used to evaluate, refine, and optimize the *nlp4sleep* algorithm for pediatric sleep phenotype extraction. Once the algorithm is refined, it will be disseminated to the remaining sites in the network. Because EHR system implementations and documentation practices for sleep-related concepts vary across institutions, each site will develop its own local gold standard dataset to further adapt and fine-tune the algorithm to achieve site-specific performance optimization. Finally, the extracted sleep phenotype concepts will be integrated with structured EHR data from each site and stored within a centralized database to support downstream analyses, such as building the machine learning models. The *nlp4sleep* system is implemented using the MedTagger NLP framework [70].

### Mental health and other assessments

Participants and caregivers will complete a battery of standardized questionnaires assessing transdiagnostic mental health symptoms, sociocultural factors, and functional outcomes (Table 3).

**Table 3:** Self- and caregiver-report questionnaires

Measure	Format	Scale
**Individual factors**		
Demographics	Researcher-administered	
Medication and treatment use	Researcher-administered	
Medical history	Researcher-administered	
Family psychiatric history	Researcher-administered	
Pubertal stage	Self-report	Petersen Pubertal Development Scale
Social media use	Self-report	Adolescents’ Digital Technology Interactions and Importance Scale
Cyber bullying	Self-report	ABCD Cyber Bully Questionnaire
Social interactions	Self-report	Self-report of Ambiguous Social Situations for Youth (SASSY)
Household income	Caregiver-report	
Sociocultural factors		
Sleep environment	Self-report	Sleep Environment Inventory
Neighborhood properties	Self- and caregiver-report	Properties of Neighborhood Scale (self- and caregiver-report)
Acculturation	Self- and caregiver-report	Acculturative Stress Inventory for Children (self-report) & Stephenson Multigroup Acculturation Scale (caregiver-report)
Discrimination	Self-report	Perceived Discrimination Scale
**Psychiatric symptoms & related measures**		
General psychopathology	Self- and caregiver-report	Youth Self Report (self-report) & Child Behavior Checklist (caregiver-report)
Psychiatric symptoms	Self- and caregiver-report	DSM-5 Cross-Cutting Symptom Measure (self- and caregiver-report)
Depressive symptoms	Self- and caregiver-report	PROMIS Emotional Distress-Depression-Pediatric Item Bank (self- and caregiver-report)
Anxiety symptoms	Self- and caregiver-report	PROMIS Pediatric Anxiety (self-report) & PROMIS Parent Proxy Anxiety (caregiver-report)
Depressive/Anxiety symptoms	Self- and caregiver-report	Revised Children’s Anxiety and Depression Scale-25 (RCADS25, self- and caregiver-report)
Mania severity	Self- and caregiver-report	7 Up (self-report) & Child Mania Rating Scale (caregiver-report)
Psychotic-like experiences	Self-report	Prodromal Questionnaire – Brief Child version (PQ-BC)
Alcohol/substance use	Self-report	CRAFFT
Impulsivity	Self-report	UPPS Short Impulse Behavior Scale
Reward sensitivity	Self-report	Behavioral Inhibition/Activation Scale (BIS/BAS)
Emotion regulation	Self-report	Difficulties in Emotion Regulation (DERS)
Repetitive Negative Thinking	Self-report	Persistent and Intrusive Negative Thoughts Scale
**Functional outcomes**		
Life satisfaction	Self- and caregiver-report	PROMIS Pediatric Life Satisfaction (self-report) & PROMIS Parent Proxy Life Satisfaction (caregiver-report)
Family relationships	Self- and caregiver-report	PROMIS Pediatric Family Relationships (self-report) & PROMIS Parent Proxy Family Relationships (caregiver-report)
Peer relationships	Self- and caregiver-report	PROMIS Peer Relationships (self-report) & PROMIS Parent Proxy Peer Relationships (caregiver-report)
Overall health	Self- and caregiver-report	PROMIS Pediatric Global Health (self-report) & PROMIS Parent Proxy Global Health (caregiver-report)
Academic performance	Self- and caregiver-report	Self-report & caregiver-report grades
Truancy	Self- and caregiver-report	Self- and caregiver-report school attendance and tardiness
Psychological stress	Self- and caregiver-report	PROMIS Psychological Stress (self-report) & PROMIS Parent Psychological Stress (caregiver-report)
Physical stress	Self- and caregiver-report	PROMIS Physical Stress (self-report) & PROMIS Parent Proxy Physical Stress (caregiver-report)

### Data collection procedures

**Fig. 2:**
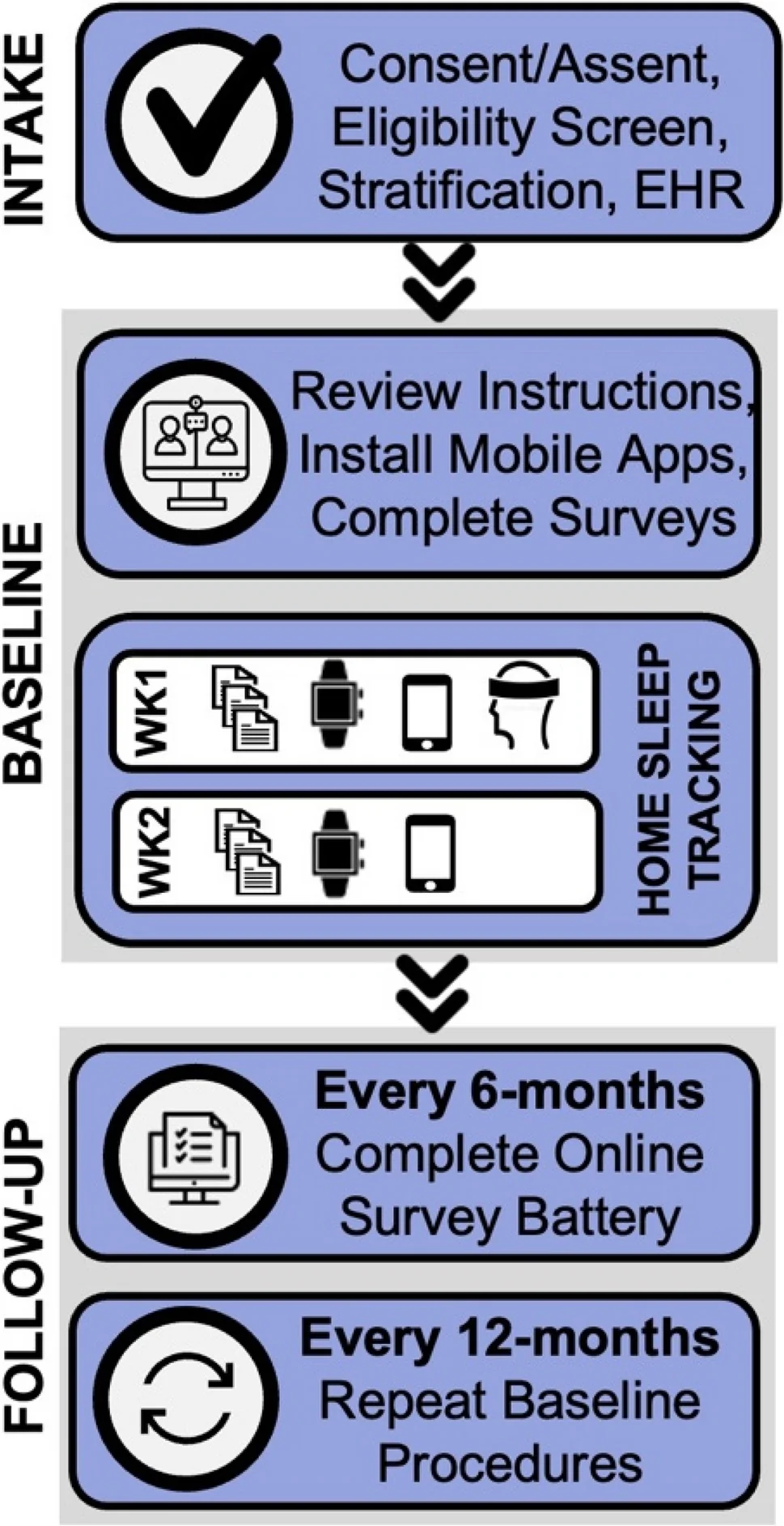
PPSN Study Flow. Schematic representation of study procedures across the intake, baseline, and follow-up phases. During intake, youth and caregivers provide consent/assent, complete eligibility screening, and review study procedures. At baseline, participants install mobile apps, complete surveys, and undergo two weeks of home-based sleep tracking using diaries, actigraphy, smartphone sensing, and up to 5 nights of wearable EEG. During the follow-up phase, participants complete online surveys every 6 months and repeat baseline procedures annually for up to three years. Data collection and adherence are monitored remotely using HIPAA-compliant platforms

Figure 2 illustrates the overall study timeline and procedures, including key activities during intake, baseline home sleep monitoring, and longitudinal follow-up.

#### Screening and consent (1 h)

An initial screening survey will establish preliminary eligibility, and can be conducted in-person, by phone or online. For online submissions, research staff will be notified upon survey completion and will review and contact families within 48 h to determine eligibility. Once eligibility is confirmed, an initial visit is scheduled. During this visit, research staff will confirm study eligibility and obtain informed consent from parents or legal guardians and assent from youth participants. Staff will review study aims and procedures, disclose potential risks, and outline the compensation schedule. After consent is obtained, research staff will guide participants through the researcher-administered surveys (see Tables 2 and 3).

#### Onboarding session (2 h)

Next, research staff will schedule an “Onboarding” session with consented participants. Prior to the Onboarding session, participants will receive study materials, including: (a) GENEActiv wrist-worn actigraphy monitor, (b) CGX Patch and charging cord, (c) 9 CGX electrode packs and a testing strip, and (d) a printed sleep monitoring packet. During the session, participants will be instructed on how to use the at-home sleep monitoring devices, including [1] daily sleep logs [2], GENEActiv actigraphy monitor [3], CGX Patch, and [4] EARS smartphone application. The sleep monitoring packet will summarize key steps and provide illustrated instructions. Staff will help participants revise their nightly routine to incorporate study procedures, encouraging use of habit-pairing strategies (e.g., applying the CGX device after brushing teeth). If researcher-administered surveys were not completed during the initial visit, they will be completed at the Onboarding session. Additionally, participants and caregivers will be provided with surveys to complete via survey link (virtual visit) or on a study-provided iPad (in-person visit).

#### Sleep monitoring (14 days)

Participants will follow a structured 14-day sleep monitoring protocol. On days 1–7, participants will wear the CGX Patch (at night) and the actigraphy watch (continuously). On days 8–14, participants will not wear the CGX Patch but will continue wearing the actigraphy watch. If participants indicate that the CGX Patch fell off or was not worn on one of the first 7 nights, they will be instructed to wear it for an additional night. Each morning and ideally within one hour of waking, participants will complete a daily sleep log, and participants with smartphones with iOS 17 and above will be notified via the EARS app to upload their screen activity from the previous day. All participants will receive a notification to complete a brief survey via the EARS app each afternoon.

#### Follow-up assessments

Follow-up assessments will occur at six-month intervals for up to three years. At each six-month assessment, self- and caregiver-report surveys will be completed; at 12-month assessments, participants will be reminded how to use the devices, and the complete survey battery and home sleep monitoring protocol will be completed.

### Ethics approval and consent to participate 

The PPSN protocol has been reviewed and approved by the University of Pittsburgh Institutional Review Board (Pitt IRB; STUDY23110103), as well as the Institutional Review Boards at Boston Children’s Hospital/Harvard Medical School and Florida International University/Nicklaus Children’s Hospital. All procedures comply with the Declaration of Helsinki and relevant national and institutional guidelines. Written informed consent will be obtained from parents or legal guardians and assent from youth participants prior to any study procedures. For EHR-based components, data will be accessed and used under IRB-approved protocols and data use agreements, and all extracted data will be handled in accordance with HIPAA regulations and local institutional policies.

### Data management and processing

All study data will be captured, stored, and managed using harmonized systems designed to ensure data security, confidentiality, and reproducibility across sites. A unique study ID assigned at enrollment will be used across all data modalities—including actigraphy, CGX Patch EEG, smartphone sensing, EHR extracts, and survey data—to enable multimodal linkage while maintaining participant confidentiality. Standard operating procedures will be shared across sites to ensure consistent data handling and preprocessing at the Pittsburgh, Miami, and Boston sites.

### Data capture and storage

Survey, clinical, and behavioral data will be collected and stored using REDCap, a secure, web-based platform protected by institutional firewalls, SSL encryption, and multi-factor authentication. Personally identifiable information and protected health information will be stored in a separate, access-restricted linkage file. All analytic datasets will be de-identified.

Actigraphy and CGX Patch data will be uploaded to secure, password-protected institutional servers and stored under the participant’s study ID. Device-level identifiers will be removed, and data will be deleted from devices following successful upload. Upon upload to secure servers, all device-derived data will be encrypted at rest according to institutional policies. Smartphone-based digital phenotyping data collected via the EARS app will be encrypted during transmission and stored in a HIPAA-compliant, access-controlled cloud environment. EHR data will be extracted through IRB-approved data requests and provided as de-identified or limited datasets, consistent with HIPAA regulations and each participating institution’s policies.

### Data processing pipelines

Standardized preprocessing pipelines will be developed for all modalities. These pipelines will include harmonization of timestamp formats, alignment of multimodal data streams, artifact detection and removal (e.g., signal loss, implausible values), and generation of derived variables (e.g., sleep duration, fragmentation indices, activity metrics, digital behavior features). Processing of unstructured EHR data will use NLP algorithms (e.g., *nlp4sleep* adapted for pediatric samples) to extract clinically relevant sleep phenotypes from narrative text; these phenotypes will be standardized and populated in structured format in the database. Computationally intensive tasks (e.g., EEG spectral decomposition, NLP feature extraction, clustering analyses) will be performed using secure high-performance computing clusters at participating institutions. All code used for data processing (e.g., R, Python, SQL) will be maintained under version control in secure, institutionally approved repositories, with Git-based workflows used to track changes and support cross-site reproducibility in collaboration with the DCC.

### Quality assurance

Quality assurance (QA) procedures will include range and logic checks, verification of data completeness, and cross-modal consistency checks (e.g., ensuring sleep logs align with actigraphy timestamps). Automated QA reports will be generated regularly to identify missing, irregular, or outlier data. Routine discrepancy resolution procedures will be used to identify and address mismatches across modalities (e.g., actigraphy–EEG misalignment, divergent sleep estimates, missing EHR fields). Training procedures and SOPs will ensure uniform data collection and processing across staff and sites.

### Coordination with the data coordinating center (DCC)

De-identified datasets will be transferred to the DCC using encrypted, institutionally approved file transfer methods. The DCC will maintain the master study database, oversee multi-site data harmonization, provide ongoing QA review, and prepare datasets for deposition into the NIMH Data Archive (NDA). The DCC will also conduct cross-site calibration checks, oversee integration of PPSN data with other IMPACT-MH studies, and support generation of NDA-compliant data structures and documentation. Data transfers will occur semi-annually or more frequently as needed during early enrollment.

### Statistical analysis

#### Aim 1: Identify and validate sleep signatures

An overview of the analytic approach is presented in Fig. 1. We define sleep signatures as empirically derived characterizations of multidimensional sleep health that can be quantified at the individual level or used to identify subgroups with similar sleep profiles. We will approach the derivation of sleep signatures from two perspectives: *signature levels* and *signature patterns*. For sleep *signature levels*, we will conduct factor analysis on data collected from actigraphy, EEG, smartphone sensing, EHR and self-report to identify latent sleep dimensions (e.g., timing, regularity, duration, satisfaction) within and across data modalities. Factor scores from each latent dimension will serve as each participant’s *signature level* on that dimension. For sleep *signature patterns*, we will use flexible clustering approaches such as multiple coalesced generalized hyperbolic distributions [71, 72] and latent class analysis to identify groups of individuals with similar within-person sleep health patterns. These approaches can address categorical and skewed data features that are common in multimodal sleep measurement [55, 71–76]. Each participant’s cluster assignment will represent their *signature pattern* of sleep health. We will also examine longitudinal change in sleep signature levels and patterns across age and pubertal development using latent trajectory [77, 78] and latent transition [79] analyses. These analyses will characterize within-person change over time in previously defined sleep signatures, providing a dynamic view of individual differences in sleep health across peri-adolescence. We will test associations between sleep signatures—and their developmental change—with transdiagnostic mental health symptomatology, using total scores on the Child Behavior Checklist [80] and Youth Self Report [81] as our primary outcomes. Secondary outcomes will include psychiatric diagnoses contained in EHR, and exploratory outcomes will include specific symptoms and functional outcomes from self-report surveys.

#### Aim 2: Develop algorithms predicting transdiagnostic mental health outcomes using sleep-circadian signatures

We will use machine learning (ML) to evaluate the predictive utility of EHR and research data—excluding sleep measures—for identifying youth at risk for worsening transdiagnostic mental health symptoms. A variety of ML models will be evaluated, including regularized regression via Elastic Net and ensemble ML (e.g., bagging, boosting, random forest).

We will then assess the incremental and combined predictive utility of sleep-related features derived from individual modalities (actigraphy, EEG, self-report, smartphone sensing). Building on Aim 1, we will next evaluate whether incorporating derived sleep signature features improves model performance. These features will include (i) signature levels, operationalized as individual factor scores indexing latent sleep dimensions across modalities, and (ii) signature patterns, operationalized as cluster assignments reflecting common multivariate sleep profiles. Stacked and agglomerative modeling approaches [82, 83], along with modern variable importance metrics [84], will be used to quantify added predictive value.

To leverage longitudinal data, we will derive summary features capturing within-person developmental change over time in sleep signatures (e.g., slopes of signature levels and transitions between signature patterns) and assess whether these features further improve predictive performance. We will additionally examine whether predictive utility varies across key sociodemographic and developmental characteristics (e.g., age, sex, race, ethnicity, pubertal status, social determinants of health) through stratified analyses and interaction testing.

To evaluate clinical translatability, we will train and test models using only EHR-derived variables (structured fields and NLP-derived features) typically available at or before a well-child visit. Best-performing models will be applied to temporally held-out EHR data from participating health systems to approximate prospective performance (e.g., discrimination, calibration) and to estimate how frequently high-risk flags would be generated in routine pediatric care. Together, these analyses provide a proof-of-concept for integrating sleep-informed features into scalable EHR-based risk detection tools.

#### Sample size and power considerations

The planned sample size of 1,200 peri‑adolescents (~ 400 per site) was selected to meet the analytic requirements of both aims while remaining feasible within three large pediatric health systems. Allowing for ~ 10% data loss, we expect ~ 1,080 participants with complete baseline data, supporting both site‑specific analyses (*N* ≈ 360) and discovery–replication splits (*N* ≈ 540).

For Aim 1, sample size justification draws on simulation studies and our prior work applying multivariate methods to multidimensional sleep data. Simulations suggest that samples of 360–540 are sufficient to extract 4–6 latent factors from 20 to 30 observed indicators with moderate-to-high communalities [85, 86], consistent with our expected parameters [19, 20, 87, 88]. Clustering studies using generalized hyperbolic mixtures and latent class models indicate that this sample range reliably recovers 3–5 subgroups [76–91], aligning with our anticipated number of distinct sleep profiles [92]. For longitudinal models (e.g., latent trajectory and transition analyses), projected retention (~ 421 participants at final follow‑up) exceeds recommended minimums for identifying discrete trajectories over four time points [90, 91, 93]. In models testing associations between sleep signatures and mental health outcomes, the anticipated sample size provides ≥ 80% power (α = 0.05) to detect odds ratios of 1.5–2.5 in site‑specific analyses and 1.2–1.4 in full‑sample analyses, assuming modest covariate correlations (*r* ≈ .20) and outcome base rates of 10–30%.

Aim 2 relies on predictive modeling rather than hypothesis testing. Here, adequacy is defined by the need for robust model training, validation, and generalization. A baseline sample of ~ 1,080 (360 per site) supports key machine learning workflows (e.g., nested cross-validation, leave-site-out testing, stacked modeling) and is consistent with prior multimodal ML studies in sleep and psychiatry [82, 83, 87].

#### Handling of missing data and site differences

We anticipate missing data due to device non-adherence, variability in EHR completeness, and attrition over the three-year follow-up. We will characterize patterns and correlates of missing data across modalities and time points. Where appropriate, we will use multiple imputation and/or inverse probability weighting to reduce bias and will fit longitudinal models using maximum likelihood estimation under missing-at-random assumptions. Site will be included as a covariate or random effect in regression and machine learning models, and models will be stratified or adjusted for key sociodemographic variables (e.g., race, ethnicity, household income) to account for differences in underlying populations and health care systems. Sensitivity analyses will compare results from complete-case samples with those obtained from imputed datasets to evaluate the robustness of findings.

## Discussion

By integrating ecologically valid, longitudinal sleep monitoring with EHR-based and survey outcomes, PPSN aims to identify developmentally sensitive sleep signatures that could be translated into scalable screening tools for PPC. Embedding sleep-informed algorithms into primary care could improve precision and equity of early risk detection and inform future efforts aimed at earlier identification and monitoring of mental health risk in peri-adolescence.

## Data Availability

PPSN data will be shared with the broader scientific community via the NIMH Data Archive (NDA) in collaboration with the Data Coordinating Center (DCC). Data will be de-identified, harmonized and formatted to comply with NDA data structure guidelines, and will be submitted to the DCC every six months for transfer to the NDA. The DCC will also support integration of PPSN with other studies participating in the Individually Measured Phenotypes to Advance Computational Translation in Mental Health (IMPACT-MH) consortium ( [https://impact-mh.org/](https:/impact-mh.org) ).
